# Supplementary material for: The Iceland-Faroe Slope Jet: a conduit for dense water toward the Faroe Bank Channel overflow
Source: Nat Commun. 2020 Oct 23;11:5390. doi: 10.1038/s41467-020-19049-5 (PMC7585422; doi:10.1038/s41467-020-19049-5)
Supplement: Supplementary file 1 — Supplementary Information [file 41467_2020_19049_MOESM1_ESM.pdf]

SUPPLEMENTARY INFORMATION

The Iceland-Faroe Slope Jet: A conduit for dense water toward the Faroe Bank Channel overflow

Stefanie Semper*[1], Robert S. Pickart[2], Kjetil Våge[1], Karin Margretha Húsgarð Larsen[3], Hjálmar Hátún[3], and Bogi Hansen[3]

1 – Geophysical Institute, University of Bergen and Bjerknes Centre for Climate Research, Bergen, Norway.
2 – Woods Hole Oceanographic Institution, Woods Hole, Massachusetts, USA.
3 – Faroe Marine Research Institute, Tórshavn, Faroe Islands.

* *Corresponding author address:* Geophysical Institute, University of Bergen and Bjerknes Centre for Climate Research, Allégaten 70, 5007 Bergen, Norway.
E-mail: stefanie.semper@uib.no

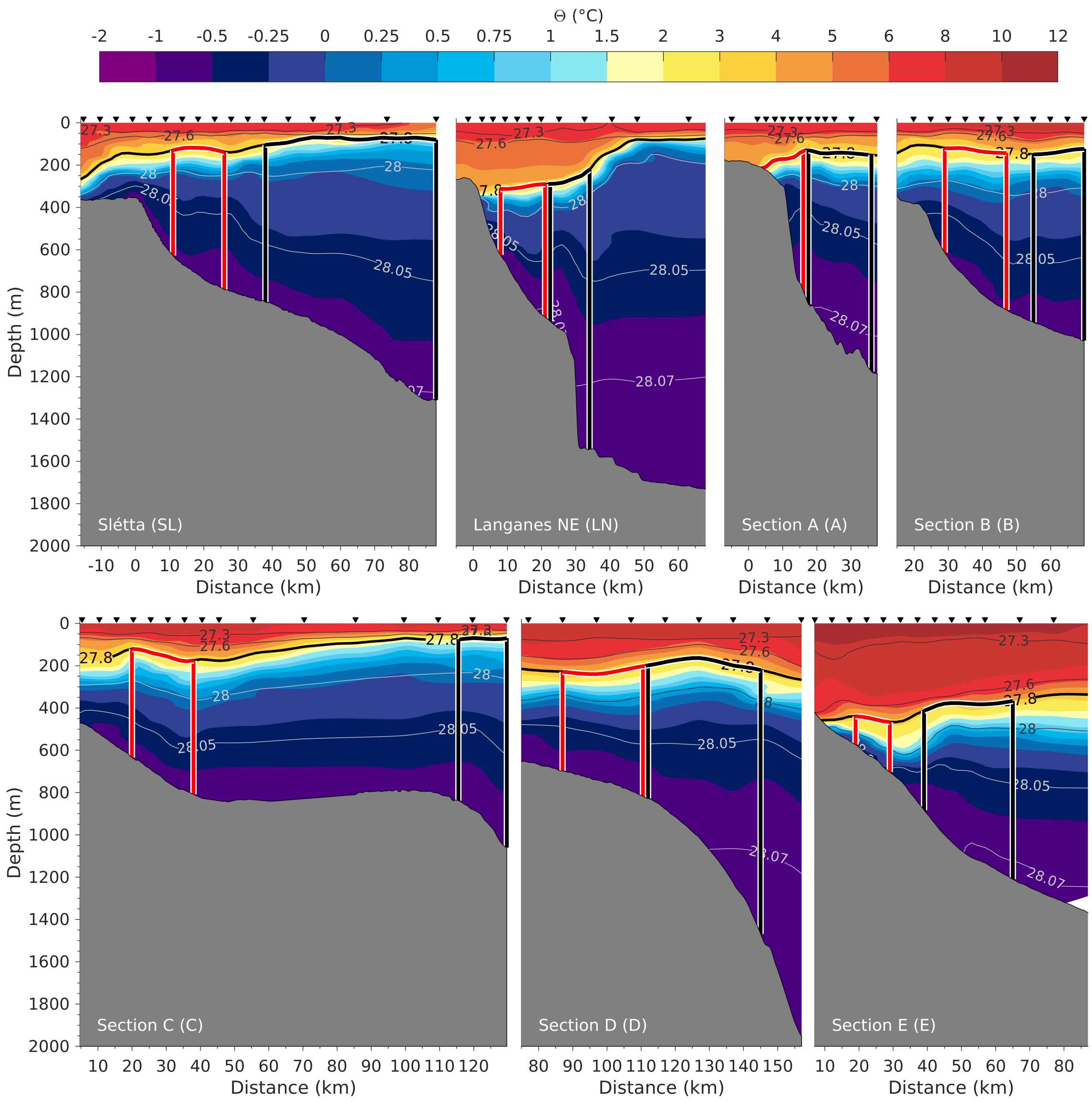

Supplementary Figure 1: Vertical sections of temperature across the IFSJ. Temperature (colour) and density (thin grey lines) for the green segments of the shipboard transects in Fig. 1b. The thick black line is the 27.8 kg m^{-3} isopycnal. The black inverted triangles indicate the locations of the stations. The red and black boxes outline the shallow and deep cores, respectively. The abbreviated names of the transects are used as labels in Fig. 1. The bathymetry is from the ship's echosounder.

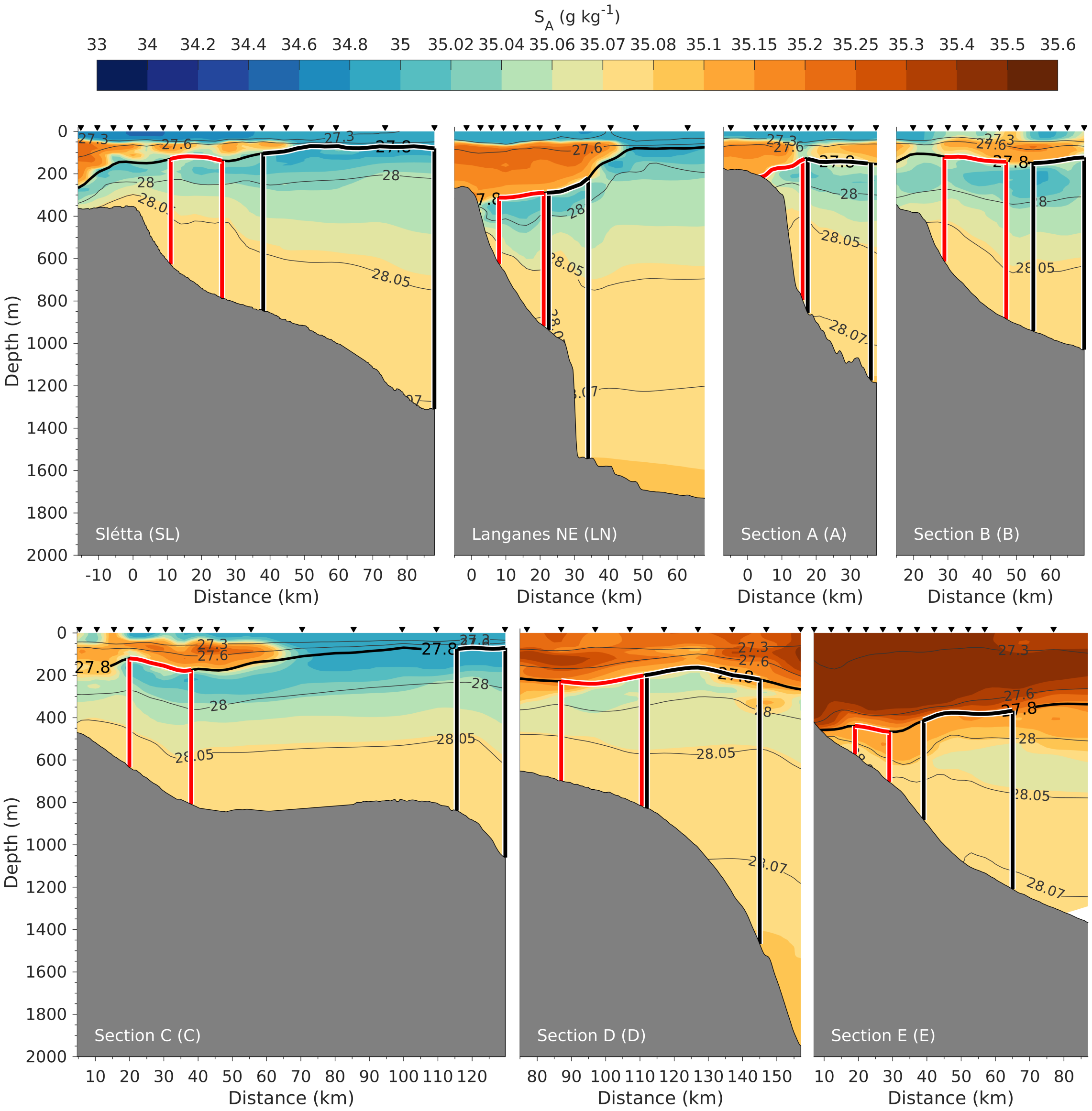

Supplementary Figure 2: Vertical sections of salinity across the IFSJ. Same as Supplementary Fig. 1 except for salinity.
